# Reversible splenial lesion syndrome associated with encephalitis/encephalopathy presenting with great clinical heterogeneity

**DOI:** 10.1186/s12883-016-0572-9

**Published:** 2016-04-18

**Authors:** Yuanzhao Zhu, Junjun Zheng, Ling Zhang, Zhenguo Zeng, Min Zhu, Xiaobin Li, Xiaoliang Lou, Hui Wan, Daojun Hong

**Affiliations:** Department of Neurology, the First Affiliated Hospital of Nanchang University, Yong Wai Zheng Street 17#, Nanchang, 330006 P. R. China; Department of Critical Care Medicine, the First Affiliated Hospital of Nanchang University, Nanchang, China; Department of Neurology, the Fourth Affiliated Hospital of Nanchang University, Nanchang, China

**Keywords:** Reversible splenial lesion syndrome, Encephalitis, Encephalopathy, Corpus callosum, Poor outcome

## Abstract

**Background:**

Reversible splenial lesion syndrome (RESLES) is a disorder radiologically characterized by reversible lesion in the splenium of the corpus callosum (SCC). Most of patients with RESLES associated with encephalitis/encephalopathy were identified in Japanese population, but almost no Chinese patients were diagnosed as RESLES associated with encephalitis/encephalopathy.

**Methods:**

Possible patients with reversible isolated SCC lesions were retrieved from January 2012 to July 2015 using keyword “restricted diffusion and isolated SCC lesion” in MRI report system from a large academic center. The clinical, laboratory and radiological data were summarized.

**Results:**

A total of 15 encephalitis/encephalopathy patients (9 males and 6 females) were identified with a reversible isolated SCC lesion. Except for 13 patients with fever symptom, 8 patients also had cold symptoms before the onset of neurological symptoms. The neurological symptoms included headache, vertigo, seizure, disturbance of consciousness, and delirious behavior. Thirteen patients completely recovered within 1 month, but 2 patients who were subjected to mechanical ventilation had persistent neurological deficits. The initial MRI features showed isolated ovoid or extending SCC lesions with homogeneous hyperintense on diffusion weighted imaging (DWI) and decreased apparent diffusion coefficient (ADC) values. The follow-up MRI revealed that isolated SCC lesions with diffuse restriction disappeared at 10 to 32 days after the initial MRI study. Fractional anisotropy map revealed the decreased value of SCC lesion in a severe case with poor prognosis.

**Conclusions:**

RESLES associated with encephalitis/encephalopathy is a reversible syndrome with an excellent prognosis in most patients, while a few patients required ventilator supporting at the early stage might have severe neurological sequelae. Reversible signal changes on DWI and ADC are identified in all patients, but fractional anisotropy values can be decreased in severe patient with neurological sequelae.

**Electronic supplementary material:**

The online version of this article (doi:10.1186/s12883-016-0572-9) contains supplementary material, which is available to authorized users.

## Background

Reversible splenial lesion syndrome (RESLES) is characterized by reversible lesion in the central portion of the splenium of corpus callosum (SCC) [1, 2]. RESLES is most often identified in patients with seizures and/or antiepileptic drugs withdrawal [3, 4]. However, it is also frequently observed in encephalitis/encephalopathy caused by various pathogens such as influenza virus, rotavirus, measles, herpesvirus 6, adenovirus, mumps, Epstein-Barr virus, *Escherischia coli*, and others [5–8]. The neurological symptoms of RESLES associated with encephalitis/encephalopathy can presented with delirious behavior, short disturbance of consciousness, and seizures, but usually had complete recovery without neurological sequelae after a short disease course [9–11]. Therefore at sometimes, RESLES associated with encephalitis/encephalopathy was interchangeably termed as clinically mild encephalitis/encephalopathy with a reversible splenial lesion (MERS) [12, 13].

Most of patients with RESLES associated with encephalitis/encephalopathy were reported in Japanese population [14, 15]. However, there were few published reports of Chinese patients diagnosed as RESLES associated with encephalitis/encephalopathy. In this study, we described the clinical data and outcomes in 15 Chinese patients with RESLES associated with encephalitis/encephalopathy identified retrospectively in order to evaluate the clinical heterogeneity of RESLES associated with encephalitis/encephalopathy.

## Methods

We performed a retrospective observational study in an academic hospital. Patients with isolated lesion in the splenium of corpus callosum visited in the first affiliated hospital of Nanchang University between January 2012 and July 2015 were screened for the study. We identified all patients by searching our MRI report system using keyword “restricted diffusion and isolated SCC lesion”. Exclusion criteria include epilepsy-related RESLES, RESLES with additional lesions in white matter, and cases without follow-up MRI. We reviewed and analyzed MRI scans and clinical charts of these patients, including information about symptoms, treatments, prognosis, electroencephalogram (EEG), and results of cerebrospinal fluid (CSF) analysis. The clinical data and radiological examinations were independently evaluated by at least two neurologists firstly. A reversible isolated SCC lesion is defined as a lesion involving the central portion of SCC without any other lesions on the first MRI, which disappears on the follow-up scanning. According to the literatures and clinician consensus, the diagnosis of encephalitis had been defined as acute onset of brain dysfunction such as acute fever, headache, seizures, delirious behavior, and disorders of consciousness with inflammatory changes such as pleocytosis of CSF [2, 16]. When patients presented with prodromal febrile illness, but without evidence of inflammatory change in central nervous system, we used the term encephalopathy [2, 16]. Fever is defined as mouth temperature more than 38 °C within the 72 h before or after presentation. The clinical procedure of etiology investigating for encephalitis includes blood, CSF, and urine cultures; CSF gram stain, India Ink stain, and modified acid-fast stain; CSF polymerase chain reaction (PCR) for herpes simplex virus 1 or 2, Epstein-Barr virus, and influenza A or B; PCR for influenza A or B in nasopharyngeal swabs; herpes simplex virus 1 (HSV1), HSV2, HSV6, cytomegalovirus, Epstein-Barr virus, and varicella-zoster virus serum IgM, and 14 d later for paired IgG antibody testing; routine human immuno-deficiency virus, treponema pallidum agglutination test, and venereal disease research laboratory test.

This study was approved by the ethics committee of the First Affiliated Hospital of Nanchang University. The written consent forms for clinical descriptions and radiological images were signed by the patients or their guardians. Herein, two cases with poor outcomes were singled out and described in detail as follows, and other patients’ descriptions were deposited in an Additional file 1.

### Case 2

**Fig. 1:**
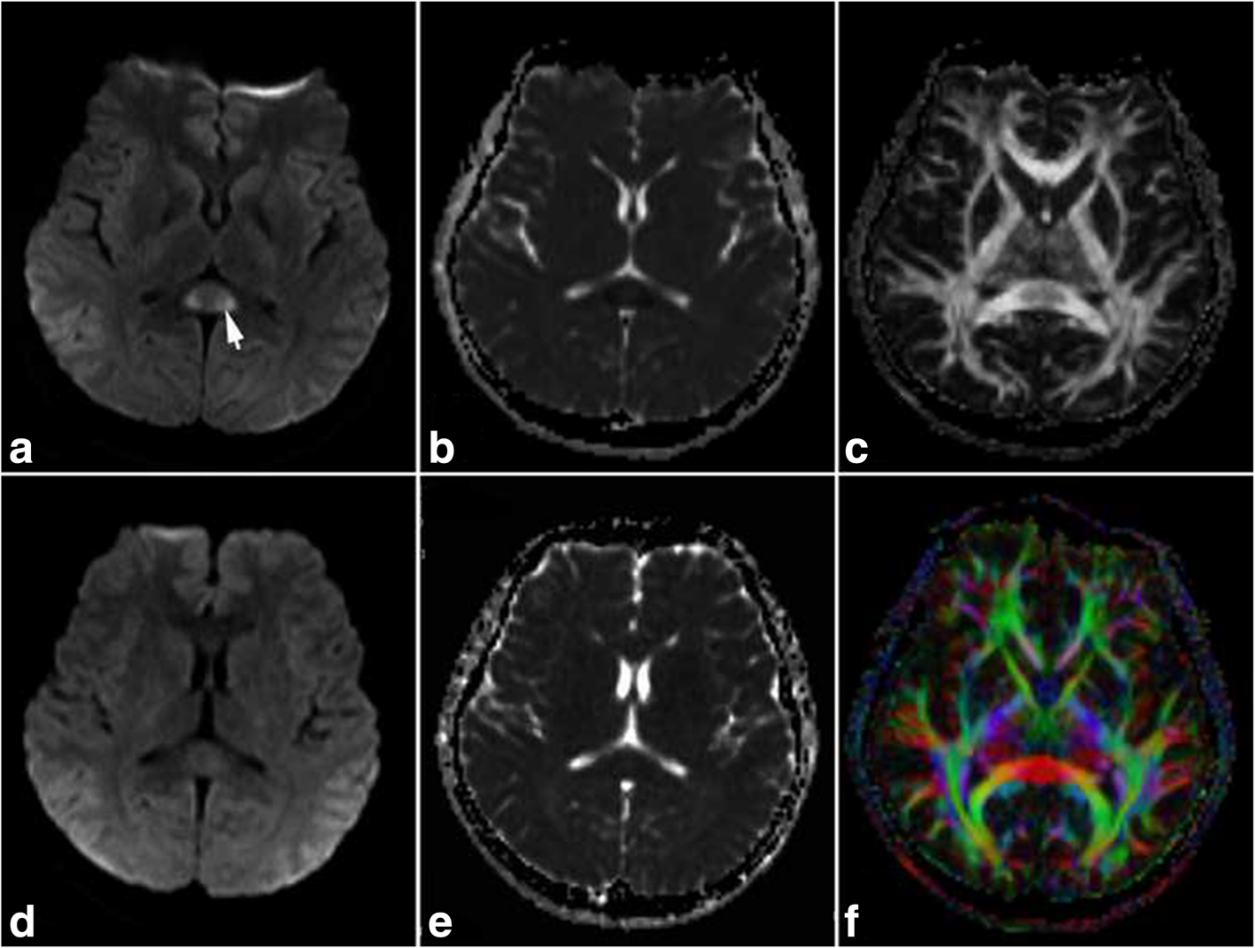
The cerebral MRI features of case 2. MRI revealed an isolated SCC lesion with obvious hyperintense on DWI (**a**, *arrow*), decreased ADC values (**b**), decreased fractional anisotropy value (**c**), and normally projecting direction of white matter fibers (**f**). At 16 days after initial MRI, second MRI showed the abnormalities of SCC nearly disappeared on DWI (**d**) and ADC (**e**)

A 23-year-old man with the complaints of fever for 6 days, disturbance of consciousness for 4 h was admitted to the hospital on March 14, 2012. The patient initially presented with cold symptoms including fever, nasal congestion, and pharyngalgia 6 days ago. At the day of admission, he had temperature up to 39 °C, severe headache, and vomiting. He suddenly lost consciousness and experienced a seizure at that night. The symptom of seizure completely stopped after 2–3 min, but coma was persistent. Physical examination on admission revealed comatose state (Glasgow Coma Scale, GCS: 7), cyanosis, stiff neck, and no voluntary movements. The emergent therapy included mechanical ventilation, antiviral (acyclovir), and antibiotics (cefoperazone). At 1 day after admission, he can detach from the ventilator, but still was in comatose state. Cerebral MRI revealed an isolated lesion in the splenium of corpus callosum with slight hyperintense on T2 weighted images (T2WI), slight hypointense on T1 weighted images (T1WI), no enhancement on contrast images, obvious hyperintense on diffusion weighted imaging (DWI) (Fig. 1a), and decreased apparent diffusion coefficient (ADC) values (Fig. 1b). Fractional anisotropy map revealed mildly decreased value of SCC lesion (Fig. 1c), but with normally projecting direction of white matter fibers (Fig. 1f). At 4 days after admission, the patient experienced several seizures again, and became deep comatose (GCS: 5). The patient was administrated with carbamazepine (400 mg/d), methylprednisolone (1000 mg/d), and intravenous immunoglobulin (IVIG) (2 g/kg). The symptoms of seizure gradually disappeared, and the temperature returned to normal limits, while disturbance of consciousness was persistent. Laboratory examination revealed serum chemistry, blood routine, thyroid function, serum ammonia, tumor biomarkers, extractable nuclei antigen (ENA) polypeptide spectrum, and anti-neutrophil cytoplasmic antibody (ANCA) were normal. A lumbar puncture revealed a mild lymphocytic pleocytosis (20 cells/μL) with normal protein and glucose contents. Oligoclonal bands were negative in serum and CSF. The quantity of myelin basic protein (MBP) was 3.8ug/L (normal <4ug/L) in CSF. Intrathecal IgG synthesis rate was 0.55 mg/24 h (normal <0.7 mg/24 h). The etiological procedure found a positive PCR for influenza A in nasopharyngeal swabs, while other extensive microbiological workups were negative. At 17 days after admission, second MRI showed the abnormalities of SCC nearly disappeared on DWI (Fig. 1d) and ADC (Fig. 1e). Spinal cord MRI was normal. At 45 days, he was transferred back to local hospital for nursing care. After 1 year follow-up, the patient was in vegetative state, nasogastric tube feeding, and bedridden.

### Case 7

**Fig. 2:**
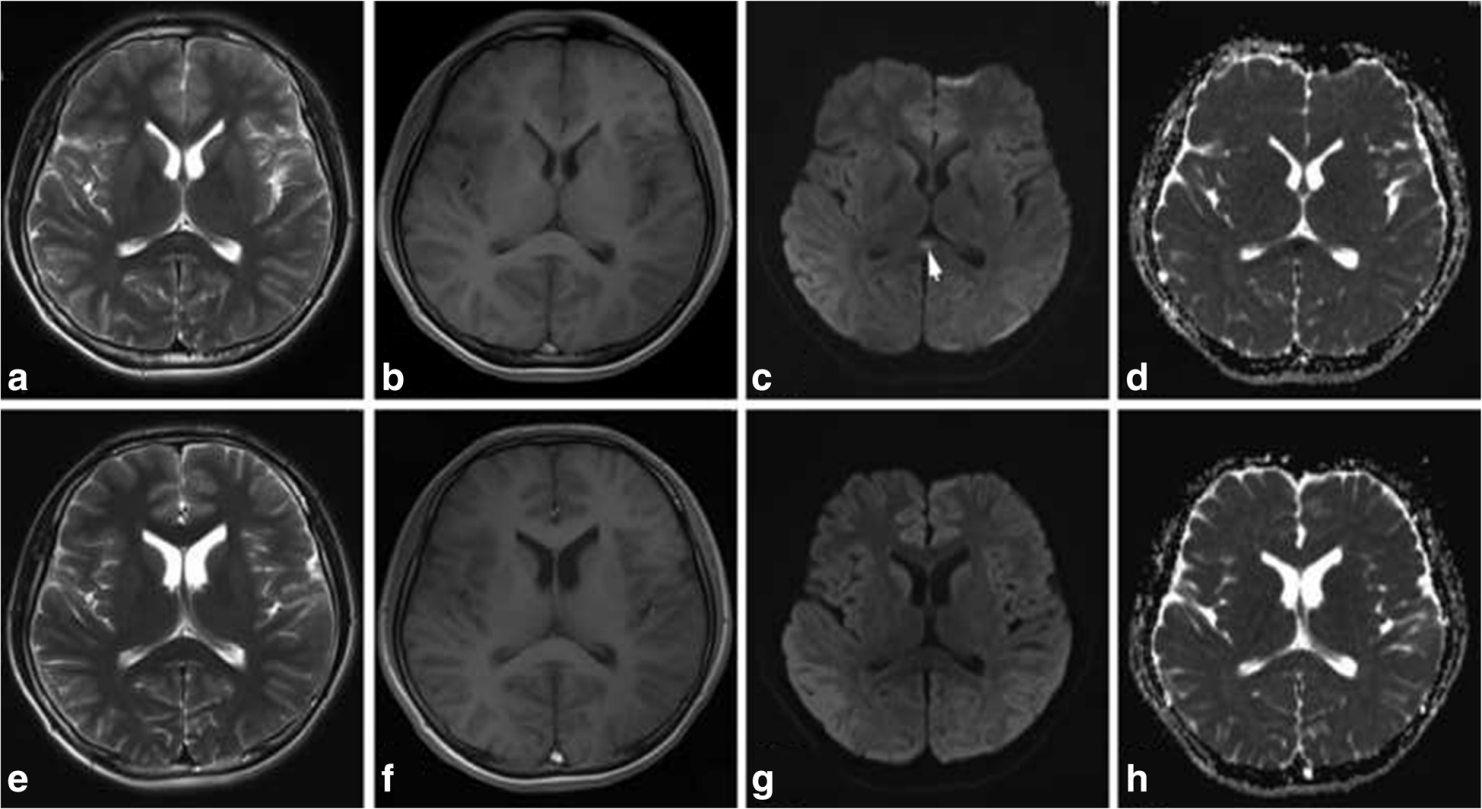
The cerebral MRI features of case 7. MRI showed an isolated SCC lesion with slight hyperintense on T2WI (**a**), slight hypointense on T1WI (**b**), hyperintense on DWI (**c**, *arrow*), and obvious decreased ADC values (**d**). At 32 days after first MRI, second MRI showed abnormal signals of the SCC completely disappearing on T2WI (**e**), T1WI (**f**), DWI (**g**) and ADC (**h**)

A 16-year-old boy with the complaints of fever and headache for 5 days, delirious behaviors for 3 days, and consciousness disturbance for 1 day was admitted to the hospital on February 12, 2014. The boy initially had fever, cough, and headache. After 3 days, the patient presented with severe headache, vomiting, and irritability, so antiviral drug (acyclovir) was administrated. After 4 days, he had consciousness disturbance, but no seizures were observed in the disease course. Physical examination on admission revealed comatose state (GCS: 8), slight limb movements responsive to pain stimulus, and negativity of bilateral pathological reflex. On admission, cerebral MRI showed an isolated lesion in the central SCC with slight hyperintense on T2WI (Fig. 2a), slight hypointense on T1WI (Fig. 2b), hyperintense on DWI (Fig. 2c), and apparent decreased ADC values (Fig. 2d). A lumbar puncture revealed a mild elevation of cell counting (15 cells/uL) with normal protein and glucose content. Oligoclonal bands were negative in serum and CSF. The quantity of MBP was 3.3ug/L in CSF. Intrathecal IgG synthesis rate was 0.61 mg/24 h. Laboratory examination revealed serum chemistry, thyroid function, serum ammonia, tumor biomarkers, ENA polypeptide spectrum, and ANCA were normal. At 1 day after admission, patients experienced more severe disturbance of consciousness, low blood pressure (70/50 mmHg), and breathing difficulty with hypoxemia and acidosis, so mechanical ventilation was administrated immediately. Meanwhile, the patient was administrated with methylprednisolone (1000 mg/d), IVIG (2 g/kg), antiviral (acyclovir), and antibiotics (vancomycin). At 10 days after admission, the patient had stable vital signs, while he was still in comatose state. At 32 days after admission, second MRI showed abnormal signals of the SCC completely disappearing on T2WI (Fig. 2e), T1WI (Fig. 2f), DWI (Fig. 2g) and ADC (Fig. 2h). After 7 months follow-up, the patient was in a condition of agrypnocoma, nasogastric tube feeding, and bedridden.

## Result

1. Clinical features
  Of the 23 patients with restricted diffusion on isolated lesion in SCC, 15 patients met the criteria for RESLES associated with encephalitis/encephalopathy and 8 patients were excluded from the study due to the following reasons: 6 had no follow-up MRI study; 1 had lesions in both SCC and bilateral white matters; 1 was associated with possible epilepsy-related RESLES due to withdrawal of antiepileptic agents. The clinical data and radiological images of 15 patients were reviewed by the co-authors together. The clinical findings of 15 patients were summarized in Table 1.

**Table 1:** Clinical data of patients with RESLES associated with encephalitis/encephalopathy

patient no.	age/sex	initial symptom	CNS manifestation (onset day)	CNS diagnosis	EEG	CSF	pathogens	therapy	prognosis (day)
1	15/M	fever	headache (2d)	encephalopathy	slow BA	normal	negative	None	CR (14d)
2	23/M	fever, nasal congestion, pharyngalgia	headache, seizure, disturbance of consciousness, meningeal irritation sign (6d)	meningoencephalitis	NE	CC: 20	influenza A	MPSL, IVIG, ACV, MV, CBZ, antibiotics,	vegetative state
3	27/F	fever, cough	headache, ataxia, slurred language (2d)	encephalopathy	NE	normal	negative	Dex, ACV	CR (9d)
4	38/M	fever, nasal congestion, cough	headache, vertigo (1d)	encephalopathy	NE	normal	herpesvirus-6	ACV	CR (11d)
5	26/M	fever, myalgias	headache, disturbance of consciousness (2d)	encephalitis	slow BA	CC:110	negative	ACV, Dex, antibiotics	CR (10d)
6	31/M	fever, pharyngalgia	vertigo (3d)	encephalopathy	slow BA	normal	negative	ACV, antibiotics	CR (20d)
7	16/M	fever, cough	headache, delirious behavior, disturbance of consciousness (2d)	encephalitis	NE	CC: 15	negative	MPSL, IVIG, ACV, MV,	agrypnocoma
8	37/F	fever, myalgias	headache, tremor (1d)	encephalopathy	normal	normal	negative	None	CR (25d)
9	25/F	fever, cough, pharyngalgia	headache, vertigo (3d)	encephalopathy	NE	normal	influenza B	ACV	CR(7d)
10	30/M	fever	limb numbness, slurred language (2d)	encephalopathy	normal	normal	negative	None	CR(12d)
11	30/F	fever, nasal congestion	headache, delirious behavior (5d)	encephalitis	normal	normal	EB virus	ACV, Dex	CR (7d)
12	13/M	fever	headache, disturbance of consciousness, seizure, meningeal irritation sign (1d)	meningoencephalolitis	slow BA	CC: 73	negative	ACV, PB, IVIG, antibiotics	CR (6d)
13	33/F	none	vertigo	encephalopathy	normal	normal	negative	None	CR (14d)
14	25/F	cough, pharyngalgia	headache (3d)	encephalopathy	NE	NE	negative	ACV	CR (10d)
15	15/M	fever	headache (1d)	encephalitis	normal	CC: 19	negative	ACV, Dex	CR (15d)

*CNS* central nervous system, *EEG* electroencephalogram, *CSF* cerebrospinal fluid, *BA* basic activity, *CR* complete recovery, *NE* not examined, *CC* cell counting (cells/uL), *MPSL* methylprednisolone, *IVIG* intravenous immunoglobulin, *ACV* acyclovir, *MV* mechanical ventilation, *CBZ* carbamazepine, *SL* slurred language, *Dex* dexamethasone, *PB* phenobarbital
  These patients included 9 males and 6 females, and their age at diagnostic time varied from 13 to 32 years (average 25.6 ± 7.98). Fever preceded neurological symptoms in 13 patients. Eight patients initially had other cold symptoms before the onset of neurological symptoms. The cold symptoms included cough (5/8), pharyngalgia (4/8), nasal congestion (3/8), and myalgias (2/8). Viral pathogens were identified in 4 of 15 patients, including influenza A or B (positive PCR in nasopharyngeal swab), herpesvirus-6 (increased IgM antibody), and Epstein-Barr virus (positive PCR in CSF). The onset of neurological symptoms ranged from day 1 to 6 of the febrile illness. The most common neurological symptom is headache (12/15), followed by disturbance of consciousness (4/15), vertigo (4/15), seizure (2/15), delirious behavior (2/15), slurred language (2/15), ataxia (1/15), tremor (1/15), and limb numbness (1/15). Two patients were treated with mechanical ventilation. Two patients had received antiepileptic drugs (phenobarbital for case 2 and carbamazepine for case 12) after the time of MRI study. Analysis of CSF revealed pleocytosis in 5 of 14 examined patients but normal glucose and protein levels. EEG showed slow basic activity characteristic of encephalitis/encephalopathy in 4 of 9 examined patients. The therapeutic regimens were variable (e.g. acyclovir for 11 patients, corticosteroids for 6 patients, and IVIG administration for 3 patients), even 4 patients were not administrated purposeful treatments. Thirteen patients clinically recovered completely within 1 month (9 patients within 2 weeks after the onset of neurological symptoms) without any neurological sequelae, but 2 patients who were required mechanical ventilation had severe neurological sequelae.
2. MRI characteristics
  In all of patients, the initial MRI study was performed within 1 week of the onset of prodromal febrile illness (Table 2). On axial images, the lesion was extended irregularly into the lateral portion of SCC in 2 patients (Fig. 1) and ovoid in the center of the SCC in the other patients (case 1, 3, 4, 5, and 7–15) (Fig. 2). There was no obvious correlation between the shape of SCC lesion and the scan date, neurological symptoms (presence or absence of seizures, date of complete recovery, with or without sequelea), or laboratory findings. The initial MRI features showed homogeneously restricted diffusion with hyperintense on DWI and decreased ADC values in SCC lesions. The lesions of SCC were homogeneously slight hyperintense on T2WI, while the lesions can be isointense to slight hypointense on T1WI. There was no enhancement of the SCC lesion after gadolinium administration in any of the 7 patients who received enhanced MRI. The SCC abnormalities of all 15 patients disappeared at follow-up MRI studies performed 10 to 32 days after the first MRI study. In at least 4 patients (case 1, 2, 7, 10), the SCC lesions disappeared before clinical symptoms reached stability or complete recovery.

**Table 2:** MRI characteristics of RESLES associated with encephalitis/encephalopathy

patient no.	initial MRI									follow-up MRI	
	scan date^1^	lesion	shape	T2WI	T1WI	Gd	DWI	ADC	DTI	scan date^2^	scan date^2^
1	day 5	SCC	ovoid	H	sL	NE	H	L	NE	day 12	none
2	day 7	SCC	extended	sH	sL	none	H	L	L	day 16	none
3	day 4	SCC	ovoid	H	L	NE	H	L	NE	day 10	none
4	day 5	SCC	ovoid	sH	I	NE	H	L	NE	day 12	none
5	day 3	SCC	ovoid	H	sL	none	H	L	NE	day 11	none
6	day 4	SCC	ovoid	sH	I	NE	H	L	NE	day 28	none
7	day 5	SCC	ovoid	sH	sL	NE	H	L	NE	day 32	none
8	day 2	SCC	ovoid	sH	I	NE	H	L	NE	day 27	none
9	day 3	SCC	ovoid	H	L	none	H	L	NE	day 14	none
10	day 3	SCC	extended	sH	sL	NE	H	L	NE	day 10	none
11	day 5	SCC	ovoid	sH	I	none	H	L	NE	day 25	none
12	day 6	SCC	ovoid	sH	sL	none	H	L	NE	day 15	none
13	day 1	SCC	ovoid	sH	I	NE	H	L	NE	day 17	none
14	day 4	SCC	ovoid	sH	sL	none	H	L	NE	day 14	none
15	day 3	SCC	ovoid	H	L	none	H	L	NE	day 16	none

*scan date* ^*1*^ from the initial symptoms, *scan date* ^*2*^from the initial MRI. *T2W* T2-weighted image, *T1WI* T1-weighted image, *Gd* gadolinium enhancement, *DWI* diffusion weighted imaging, *ADC* apparent diffusion coefficient, *DTI* diffusion tensor imaging, *SCC* splenium of the corpus callosum, *H* high intensity, *L* low intensity, *I* isointensity, *sH* slight high intensity, *sL* slight low intensity, *NE* not examined

## Discussion

In this study, we described the clinical characteristics and outcomes in 15 patients with RESLES associated with encephalitis/encephalopathy in mainland China. These patients initially presented with febrile illness, and then had CNS manifestations after several days. Due to the limits of pathogens and CSF examinations, 6 patients can be considered as encephalitis, while 9 other patients can be only diagnosed as infection-associated encephalopathy [2, 14]. However, all patients had a complete radiological regression of the isolated SCC lesion within several weeks.

Almost all patients with RESLES could be complete recovery without neurological sequelae after the acute disease course [1, 2]. Therefore, RESLES associated with encephalitis/encephalopathy was also known as a type of clinically mild encephalitis/encephalopathy [2, 14]. However, we found two patients (case 2 and 7), who required ventilator supporting in the acute period of disease course, had persistent comatose state and poor prognosis, even though the abnormal lesions disappeared within 1 month. Through detailed clinical investigations, the typical evidences of acute disseminated encephalomyelitis (ADEM), multiple sclerosis (MS), autoimmune encephalitis, vasculitis of central nervous system, and various metabolic or toxic encephalopathies were not found in the two patients. Although some rare underlying disorders can not be completely excluded, the clinical pictures of the two patients conformed to the diagnosis of RESLES associated with encephalitis/encephalopathy. Therefore, RESLES associated with encephalitis/encephalopathy should not be simply considered as a mild clinic-radiological disorder with an excellent prognosis, particularly in patients required ventilator supporting in acute stage of disease course.

Due to the insufficient number of patients, there had been no evidence-based therapeutic regimen for RESLES associated with encephalitis/encephalopathy. According to the descriptions of literatures, the commonest prescriptions included acyclovir, antibiotics, anti-epileptic drugs, corticosteroids, and IVIG [2, 17]. We also found that the treatment can change from one case to other case in our observational patients; even 4 cases with good prognosis had not been administrated any purposeful treatments.

The pathogenesis of the selective splenial lesion is not fully understood in RESLES associated with encephalitis/encephalopathy. Multiple etiologies are considered involvements into the splenial lesion [2, 18, 19]. Therefore, as a distinct radiological spectrum disorder, cerebral MRI still is the first choice to identify lesions of the SCC, particularly to detect early changes, and helps interpret the nature of the lesion [1]. The SCC lesions consistently showed a homogeneous and reversible pattern on DWI, differentiating these lesions from persistent ischemia, where ADC reduction with subsequent reversal is uncommon. Our cases showed reversible DWI and ADC reduction supporting that cytotoxic edema of the SCC is an important mechanism in this condition. Interestedly, we found the SCC lesion presented with decreased fractional anisotropy values in the initial MRI scanning, which was different from the findings described by other cases [20, 21]. The decreased fractional anisotropy values in areas of diffusion restriction may indicate the disarrangement of water molecule motivation along the axonal fibers, which also may indicate the destruction of bridge architecture associated with neurological deficits in the patient. However, the clinical significance between fractional anisotropy value and prognosis should be further investigated in more cases. In addition, some cases with slightly tinny hyperintense on T2 and DWI had severe clinical course and poor outcomes, but other cases with obvious hyperintense on T2 and DWI presented with slight clinical course. The phenomenon might indicate that the intensity and formation of SCC lesion were not closely associated with the clinical features.

Since a few patients with ADEM can present with isolated SCC lesion in early stage of disease course [22], ADEM should be a differential diagnosis of RESLES associated with encephalitis/encephalopathy. ADEM is an immune inflammatory disorder, which is clinically characterized as seizures, focal neurological signs, disturbance of consciousness, and recovery after immunomodulatory therapy. MRI in ADEM usually shows multiple foci of T1 and T2 prolongation with enhancement in bilateral or asymmetric subcortical white matters [21]. Therefore, we excluded a case with lesions both SCC and bilateral white matters in order to obtain a more strict clinical diagnosis, although some patients with RESLES associated with encephalitis/encephalopathy were well described with additional white matter lesions [23]. The lesions of ADEM usually evolve over weeks to months and disappear only after several months [21]. However, our patients developed neurological symptoms quickly after the onset of febrile illness. The SCC lesions in these patients had no contrast enhancement, and most of them disappeared completely within month. Therefore, the SCC lesions in our patients are clinically and radiologically different from those of ADEM. Autoimmune encephalitis such as anti-N-methyl-D-aspartate (NMDA) receptor encephalitis also should be differentiated [24]. Corpus callosum can be affected in very few patients with anti-NMDA receptor encephalitis, while the lesions were not quickly reversible diffusion restriction with reduced apparent diffusion coefficient in patients who usually were young female with ovarian teratoma [25].

The major limitations of our work were its retrospective nature and relatively small sample size. A larger prospective study with long-term follow-up would be necessary to corroborate our findings.

## Conclusion

RESLES associated with encephalitis/encephalopathy is a distinct disorder, which usually affects children or young adults with febrile illness. Although RESLES associated with encephalitis/encephalopathy is a reversible syndrome with an excellent prognosis in most patients, a few patients required ventilator supporting at the early stage might have severe neurological sequelae. Reversible signal changes on DWI and ADC are identified in all of patients, but fractional anisotropy values can be decreased in severe patient with neurological sequelae.

### Ethics and consent to participate

This study was approved by the ethics committee of the First Affiliated Hospital of Nanchang University.

### Consent to publish

Written consent for publication had obtained from patients or their parents.

### Availability of data and materials

The detailed descriptions about those patients can be available in a Additional file 1.

## Additional file

Additional file 1: Reversible splenial lesion syndrome associated with encephalitis/encephalopathy presenting with great clinical heterogeneity. (52 kb)

## Abbreviations

RESLES: Reversible splenial lesion syndrome
SCC: splenium of the corpus callosum
MRI: magnetic resonance image
EEG: electroencephalogram
CSF: cerebrospinal fluid
PCR: polymerase chain reaction
T2WI: T2 weighted images
T1WI: T1 weighted images
DWI: diffusion wighted imaging
ADC: apparent diffusion coefficient
IVIG: intravenous immunoglobulin
ENA: extractable nucler antigen
ANCA: anti-neutrophil cytoplasmic antibody
MBP: myelin basic protein
ADEM: acute disseminated encephalomyelitis
MS: multiple sclerosis
NMDA: N-methyl-D-aspartate
