# Supplementary material for: Reversible splenial lesion syndrome associated with encephalitis/encephalopathy presenting with great clinical heterogeneity
Source: BMC Neurol. 2016 Apr 18;16:49. doi: 10.1186/s12883-016-0572-9 (PMC4835842; doi:10.1186/s12883-016-0572-9)
Supplement: Additional file 1: — Reversible splenial lesion syndrome associated with encephalitis/encephalopathy presenting with great clinical heterogeneity. (52 kb) [file 12883_2016_572_MOESM1_ESM.doc]

**Additional supporting file**

**Reversible splenial lesion syndrome associated with encephalitis/encephalopathy presenting with great clinical heterogeneity**

Yuanzhao Zhu^1^, Junjun Zheng^1^, Ling Zhang^1^, Zhenguo Zeng^2^, Min Zhu^1^, Xiaobin Li^1^, Xiaoliang Lou^3^, Hui Wan^1^, Daojun Hong^1^

1. Department of Neurology, the First Affiliated Hospital of Nanchang University, China
2. Department of Critical Care Medicine, the First Affiliated Hospital of Nanchang University, China
3. Department of Neurology, the Fourth Affiliated Hospital of Nanchang University, China

Email address: Zhu Y: 846366855@qq.com; Zheng J: zjj259@163.com; Zhang L: 601944681@qq.com; Zeng Z: zzg6501@163.com; Zhu M: zhumin1@126.com; Li X: lxb0203208@126.com; Lou X: 895936295@qq.com; Wan H: ncwanhui@sina.com; Hong D: hongdaojun@hotmail.com

**Corresponding author:**

Dr. Daojun Hong

Department of Neurology, The First Affiliated Hospital of Nanchang University

Yong Wai Zheng Street 17^#^, Nanchang, 330006, P.R.China

Telephone: 86-791-8869-2511; Fax number: 86-791-8869-2511

E-mail: hongdaojun@hotmail.com

**Case 1:** A 15-year-old boy was admitted to our medical center for severe headache on February 1st 2012. He had fever (highest 39℃), bilateral temporal headache, nausea, and vomiting two days ago. The community physician administrated hydration therapy, and then the patient's temperature became normal, but headache was still persistent and got worse. Cerebral CT was negative. Therefore, cerebral MRI was performed, and revealed an isolated long T2 abnormal signal accompanying with obvious hyperintense on diffusion weighted imaging (DWI), and decreased apparent diffusion coefficient (ADC) values in the splenium of corpus callosum (SCC) at the third day after admission. Physical examination on admission revealed no abnormal signs. Laboratory examination revealed serum chemistry, blood routine, thyroid function, serum ammonia, tumor biomarkers, extractable nuclei antigen (ENA) polypeptide spectrum, and anti-neutrophil cytoplasmic antibody (ANCA) were normal. The battery of examinations on cerebrospinal fluid (CSF) was normal. The extensive etiological procedure was negative. Electroencephalogram revealed a slow basic activity, but no epileptiform waves were identified. The patient was given hydration and multiple vitamins treatment, and the headache symptom was complete recovery two weeks later. At 15 days after admission, second MRI showed the abnormalities of SCC completely disappeared on DWI and ADC. After 3 months follow-up, the patient was complete intact.

**Case 3:** A 27-year-old woman was admitted to our medical center for acute ataxia symptoms on June 22nd 2013. She had fever and cough 5 days before admission. The temperature was not constant with high fever than noon, and the highest temperature reached 39.8℃. She presented with headache, slurred speech, and unsteady gait 3 days ago. No nausea, vomiting, hemiplegia, and disturbance of consciousness. Neurological examination indicated limb ataxia and slurred speech. Cerebral CT was negative. Cerebral MRI at 1 day before admission revealed isolated hypertense T2 signal, hypotense T1 signal, hypersignal on DWI, and reduced ADC values in the central SCC. The routine CSF tests were normal. Laboratory examination revealed serum chemistry, blood routine, thyroid function, serum ammonia, tumor biomarkers, ENA polypeptide spectrum, and ANCA were normal. The extensive etiological procedure was negative. The patient was given dexamethasone and acyclovir treatment, and the symptoms were complete recovery 9 days after admission. At 9 days after admission, second MRI showed the abnormalities of SCC nearly disappeared on DWI and ADC. After 6 months follow-up, the patient was totally healthy.

**Case 4:** A previously healthy 38-year-old man referred to our hospital on December 15th 2013 with the complaints of vertigo repeatedly for 1 day. Before the vertigo symptoms, the patient had cold symptoms including fever, cough, and nasal congestion, but without vomiting and tinnitus. Neurological examination was intact. At the fifth day after initial symptoms, cerebral MRI demonstrated an isolated lesion in the SCC with hyperintense on T2, isointense on T1, hyperintense on DWI, and decreased ADC. Serum viral antibody tests revealed an increased IgM antibody of herpesvirus-6, while other extensive microbiological workups were negative. Laboratory examination revealed serum chemistry, blood routine, thyroid function, serum ammonia, tumor biomarkers, ENA polypeptide spectrum, and ANCA were normal. The routine CSF tests were normal. The patient was given acyclovir drip for 2 weeks, and the symptoms were complete recovery 11 days after admission. At 11 days after admission, second MRI showed the abnormalities of SCC completely disappeared on DWI and ADC. After 3 months follow-up, the patient was complete intact.

**Case 5:** A 26-year-old male patient was admitted to our hospital for acute disturbance of consciousness on February 3rd 2014. He had fever (highest 38.9℃) and myalgias for 4 days before admission, and presented with severe headache and disturbance of consciousness 2 days later. Physical examination revealed stupor state, but limbs motor was responsive to stimulus. Cerebral CT was negative. At the second day after neurological symptoms, cerebral MRI demonstrated an isolated lesion in the SCC with hyperintense on T2, hypointense on T1, hyperintense on DWI, and decreased ADC. The lesion was not enhanced in contrasted MRI. Electroencephalogram revealed a slow basic activity. CSF examinations revealed a lymphocytic pleocytosis (110 cells/μL) with normal other routine tests. Blood routine revealed a high level of white cells. Laboratory examination revealed serum chemistry, thyroid function, serum ammonia, tumor biomarkers, ENA polypeptide spectrum, and ANCA were normal. The extensive etiological procedure was negative. The patient was given dexamethasone, acyclovir, and antibiotics treatment, and the symptoms were complete recovery 10 days after admission. At 12 days after admission, second MRI showed the abnormalities of SCC disappeared on DWI and ADC. After 6 months follow-up, the patient was complete intact.

**Case 6:** A 31-year-old male patient was admitted to our hospital on February 11th 2014. He initially presented with mild fever and pharyngalgia without cough and nasal congestion. Three days later, the patient suddenly endured vertigo accompanied with nausea, but without headache, limb numbness, and disturbance of consciousness. Cerebral CT was normal. At the fifth day after initial symptoms, cerebral MRI demonstrated an isolated lesion in the SCC with hyperintense on T2, isointense on T1, hyperintense on DWI, and decreased ADC. The routine CSF tests were normal. Laboratory examination revealed serum chemistry, thyroid function, serum ammonia, tumor biomarkers, ENA polypeptide spectrum, and ANCA were normal. The extensive etiological procedure was negative. Electroencephalogram revealed a slow basic activity. The patient was given acyclovir and antibiotics treatment, and the symptoms were complete recovery 17 days after admission. At 4 weeks later, second MRI showed the abnormalities of SCC disappeared on DWI and ADC. After 6 months follow-up, the patient was complete intact.

**Case 8:** A 37-year-old female patient was admitted to our hospital on February 23rd 2014. She had fever (highest 39.2℃) and myalgias for 2 day before admission, and presented with bilateral temporal headache and limb tremor 1 day later. Physical examination was intact. At the second day after neurological symptoms, cerebral MRI demonstrated an isolated lesion in the SCC with hyperintense on T2, isointense on T1, hyperintense on DWI, and decreased ADC. The routine CSF tests were normal. Laboratory examination revealed serum chemistry, blood routine, thyroid function, serum ammonia, tumor biomarkers, ENA polypeptide spectrum, and ANCA were normal. The extensive etiological procedure was negative. Electroencephalogram revealed a slow basic activity. The patient was given hydration and multiple vitamins treatment, and the headache and tremor symptoms were complete recovery 3 weeks later. At 25 days, second MRI showed the abnormalities of SCC nearly disappeared on DWI and ADC. After 5 months follow-up, the patient was complete intact.

**Case 9**: A 25-year-old woman with complaints of fever and headache for 5 days was admitted to the hospital on June 16, 2014. The patient initially presented cold symptoms including fever, cough, pharyngalgia, and headache. When she had more severe headache with vertigo at 5 days after onset, she was transferred to our hospital for emergent evaluation. Physical examination on admission revealed no abnormalities of neurological signs. Cerebral MRI showed an isolated lesion in the central SCC with hyperintense on T2WI, significant hyperintense on DWI, decreased ADC values, and no enhancement of the lesion. A lumbar puncture revealed normal cell counting, protein and glucose contents. Laboratory work-up was in the normal range. The etiological procedure found a positive PCR for influenza B in nasopharyngeal swabs, while other extensive microbiological workups were negative. Patient was treated with acyclovir and painkillers, and the clinical symptoms were completely recovered at 4 days after admission. A follow-up brain MRI was performed 14 days after initial MRI and showed complete recovery of the lesion.

**Case 10:** A 30-year-old male patient was admitted our hospital on July 14th 2014. He had fever (highest 38.8℃) for 3 days without any cold symptoms, and then felt numbness in bilateral lower limbs accompanied with slurred language 2 days later. Physical examination on admission revealed no abnormalities of neurological signs except for slurred language. Cerebral CT was negative. At the second day after neurological symptoms, cerebral MRI demonstrated an isolated extended lesion in the SCC with hyperintense on T2, hypointense on T1, hyperintense on DWI, and decreased ADC. The routine CSF tests were normal. Laboratory examination revealed serum chemistry, blood routine, thyroid function, serum ammonia, tumor biomarkers, ENA polypeptide spectrum, and ANCA were normal. The extensive etiological procedure was negative. Electroencephalogram revealed a slow basic activity. The patient was given multiple vitamins, and the neurological symptoms were complete recovery 12 days later. At the 10th day after admission, second MRI showed the abnormalities of SCC disappeared on DWI and ADC. After 3 months follow-up, the patient was complete intact.

**Case 11:** A 30-year-old female patient was admitted our hospital on July 15th 2014. She had fever and nasal congestion for 5 days, and then suddenly presented with severe headache and delirious behavior. Physical examination on admission revealed agitation and no cooperation for examination, but had voluntary limb motilities and clear speeches. At the second day after neurological symptoms, cerebral MRI demonstrated an isolated lesion in the SCC with hyperintense on T2, isointense on T1, hyperintense on DWI, and decreased ADC. No abnormalities were observed in the enhanced MRI. The routine CSF tests were normal. CSF viral PCR tests revealed a positivity of Epstein-Barr virus, while other extensive microbiological workups were negative. Laboratory examination revealed serum chemistry, thyroid function, serum ammonia, tumor biomarkers, ENA polypeptide spectrum, and ANCA were normal. Electroencephalogram revealed a slow basic activity. The patient was given dexamethasone and acyclovir, and the neurological symptoms were complete recovery 1 week later. At 25 days later, second MRI showed the abnormalities of SCC disappeared on DWI and ADC. After 6 months follow-up, the patient was complete intact.

**Case 12:** A 13-year-old boy was emergently admitted in our hospital for seizure on August 25th 2014. The boy initially had fever (38.5℃) 1 day, and then he presented with severe headache and irritability. When the patient was admitted for several hours, he suddenly lost consciousness and experienced a seizure. The symptom of seizure completely stopped after 2-3 minutes, but was still unconsciousness. Physical examination revealed stupor, stiff neck, and no voluntary movements. At the 5th day after admission, cerebral MRI showed an isolated lesion in the central SCC with slight hyperintense on T2WI, slight hypointense on T1WI, hyperintense on DWI, and apparent decreased ADC values. The lesion was not contrasted on enhanced MRI. A lumbar puncture revealed an elevation of cell counting (73 cells/uL) with normal protein and glucose content. Oligoclonal bands were negative in serum and CSF. The quantity of MBP was 2.7 ug/L in CSF. Intrathecal IgG synthesis rate was 0.48 mg/24h. Blood routine indicated elevation of the counting of white cells. Laboratory examination revealed serum chemistry, thyroid function, serum ammonia, tumor biomarkers, ENA polypeptide spectrum, and ANCA were normal. Extensive etiological investigations were negative. Electroencephalogram revealed a slow basic activity. The patient was administrated with IVIG (2g/kg), antiviral (acyclovir), antibiotics (vancomycin), and phenobarbital. The neurological symptoms were complete recovery 6 days later. At 3 weeks later, second MRI showed the abnormalities of SCC completely disappeared on DWI and ADC. After 6 months follow-up, the patient was complete intact.

**Case 13:** A 33-year-old woman was admitted our hospital for vertigo on January 19th 2015. She felt sudden vertigo without vomiting and tinnitus. No nausea, speech problems, hemiplegia, and disturbance of consciousness. Physical examination on admission revealed no neurological symptoms. Cerebral MRI demonstrated an isolated lesion in the SCC with hyperintense on T2, isointense on T1, hyperintense on DWI, and decreased ADC. The routine CSF tests were normal. Extensive microbiological workups were negative. Laboratory examination revealed serum chemistry, blood routine, thyroid function, serum ammonia, tumor biomarkers, ENA polypeptide spectrum, and ANCA were normal. Electroencephalogram was normal. The patient was given multiple vitamins, and the neurological symptoms were complete recovery 2 weeks later. At 17 days later, second MRI showed the abnormalities of SCC disappeared on DWI and ADC. After 3 months follow-up, the patient was complete intact.

**Case 14:** A 25-year-old female patient was admitted our hospital for headache on March 9th 2015. She had cough and fever and pharyngalgia 4 days ago, and then had severe headache 1 day ago. Physical examination on admission revealed no abnormalities of neurological symptoms. Cerebral MRI demonstrated an isolated extended lesion in the SCC with hyperintense on T2, hypointense on T1, hyperintense on DWI, and decreased ADC. No abnormalities were observed in the enhanced MRI. Extensive microbiological workups were negative. Laboratory examination revealed serum chemistry, blood routine, thyroid function, serum ammonia, tumor biomarkers, ENA polypeptide spectrum, and ANCA were normal. The patient was given acyclovir, and the headache was complete recovery 10 days later. At 2 weeks later, second MRI showed the abnormalities of SCC disappeared on DWI and ADC. After 6 months follow-up, the patient was complete intact.

**Case 15:** A 15-year-old boy was admitted our hospital on July 5th 2015. He had fever (highest 39.1℃) 1 day ago, and then suddenly presented with severe headache. Physical examination on admission revealed no signs of neurological involvements. At the admission day, cerebral MRI demonstrated an isolated extended lesion in the SCC with hyperintense on T2, hypointense on T1, hyperintense on DWI, and decreased ADC. No abnormalities were observed in the enhanced MRI. Extensive microbiological workups were negative. Laboratory examination revealed serum chemistry, blood routine, thyroid function, serum ammonia, tumor biomarkers, ENA polypeptide spectrum, and ANCA were normal. Electroencephalogram was no abnormalities. The patient was given dexamethasone and acyclovir, and the neurological symptoms were complete recovery a half of month later. At 16 days later, second MRI showed the abnormalities of SCC disappeared on DWI and ADC. After 6 months follow-up, the patient was complete intact.
